# Reimagine the ICU: Healthcare Professionals’ Perspectives on How Environments (Can) Promote Patient Well-Being

**DOI:** 10.1177/19375867231219029

**Published:** 2024-01-31

**Authors:** Chan Mi Kim, Esther M. van der Heide, Thomas J. L. van Rompay, Geke D. S. Ludden

**Affiliations:** 1 Department of Design, Production, and Management, Faculty of Engineering Technology, University of Twente, Enschede, the Netherlands; 2 Patient Care and Monitoring Department, Philips Research, Eindhoven, the Netherlands; 3 Department of Communication Science, Faculty of Behavioral, Management, and Social Sciences, University of Twente, Enschede, the Netherlands

**Keywords:** co-creation, healing environment, intensive care, mixed-method study, patient experience, patient well-being, strategy, technology

## Abstract

**Objective:**

This study aims (1) to understand the needs and challenges of the current intensive care unit (ICU) environments in supporting patient well-being from the perspective of healthcare professionals (HCPs) and (2) to explore the new potential of ICU environments enabled by technology.

**Background:**

Evidence-based design has yielded how the design of environments can advocate for patient well-being, and digital technology offers new possibilities for indoor environments. However, the role of technology in facilitating ICU patient well-being has been unexplored.

**Method:**

This study was conducted in two phases. First, a mixed-method study was conducted with ICU HCPs from four Dutch hospitals. The study investigated the current environmental support for care activities, as well as the factors that positively and negatively contribute to patient experience. Next, a co-creation session was held involving HCPs and health technology experts to explore opportunities for technology to support ICU patient well-being.

**Results:**

The mixed-method study revealed nine negative and eight positive patient experience factors. HCPs perceived patient emotional care as most challenging due to the ICU workload and a lack of environmental support in fulfilling patient emotional needs. The co-creation session yielded nine technology-enabled solutions to address identified challenges. Finally, drawing from insights from both studies, four strategies were introduced that guide toward creating technology to provide holistic and personalized care for patients.

**Conclusion:**

Patient experience factors are intertwined, necessitating a multifactorial approach to support patient well-being. Viewing the ICU environment as a holistic unit, our findings provide guidance on creating healing environments using technology.

## Introduction

Staying in the intensive care unit (ICU) is known to be highly stressful and traumatic for patients due to their critical condition as well as medical procedures, pain, and a disturbing environment (Egerod et al., 2015). Stressful ICU experiences negatively affect patients’ health outcomes during and after an ICU stay. Patients with stressful ICU experiences are more likely to develop delirium (Zaal et al., 2013), stay longer in the ICU (Gruenberg et al., 2006; May et al., 2021), and suffer from cognitive impairment (Davydow et al., 2013) and postintensive care syndrome (Granja et al., 2005; Parker et al., 2015) significantly reducing quality of life (Maley et al., 2016; Oeyen et al., 2010). Next to that, studies (Goldfarb et al., 2017; Nielsen et al., 2019; Ulrich et al., 2004) have shown that positive ICU experiences result in better health outcomes. These results highlight the need to promote patient experience and, more specifically, patient well-being in the ICU as a means to support healing and recovery.

Well-being in general refers to the state of individuals where they feel happy, healthy, and satisfied with their life (Ryan & Deci, 2001). The concept of well-being, therefore, goes beyond the traditional biomedical view of health which is considered as the absence of disease; it comprises various dimensions of an individual’s life including their physical, mental, social, and environmental status (Kiefer, 2008; Ryan & Deci, 2001). For instance, a person with a leg impairment could still experience well-being as long as personal values, such as a sense of autonomy or flourishing, are fulfilled. Taking this holistic view, well-being is relevant and achievable in the context of critical care, provided that patients are in an environment that supports what is important for their well-being.

The understanding that the environment has an effect on the healing and well-being of patients sparked the design of healing environments. A healing environment refers to the entire context surrounding patients that promotes health and well-being by catering to their physical, mental, social, and spiritual needs (Sakallaris et al., 2015). To effectively design healing environments, evidence-based design (EBD) approaches have been used: empirical evidence on how the design of physical environments can promote better clinical outcomes. EBD has been applied to various environmental factors ranging from space layout to sound and natural light in the space that affect physical (e.g., injuries, effectiveness, infection, sleep) as well as psychological (e.g., stress, privacy, satisfaction) components (Sakallaris et al., 2015).

A relatively new development in this area is the use of digital technology. This has expanded and diversified the role of environments in supporting patient well-being. For instance, an interactive immersive projection technology extends what physical surfaces of the environment can offer beyond color and texture. It creates dynamic visual stimulation that can change over time and, hence, can accommodate changing emotional needs of patients by providing stimuli that evoke high or low arousal (Maltha, 2022). The role of sound in healing environments can go beyond noise reduction by actively comforting patients through positive sound tones conveying messages from loved ones (Özcan et al., 2021). As such, digital technology opened up new avenues for EBD in healing environments. Previous review papers have outlined the implications of technology in supporting patients’ healing and well-being (Cox & Curtis, 2016; Kim et al., 2021; Silvera-Tawil et al., 2020; Smits et al., 2022). However, the new potential of designing healing environments facilitated by technology remains largely unexplored. To optimize the environmental support in the ICU, obtaining a comprehensive view of the fundamental care needs of patients and HCPs in the ICU is important. Several studies looked into ICU patient experiences, of which most studies focus on barriers to patient experiences (Abuatiq, 2015; Krampe et al., 2021; Zengin et al., 2020) and some include also facilitators (Jakimowicz et al., 2017; Samuelson, 2011). In both cases, there was only limited focus on the influence of environmental factors. Furthermore, most of these studies derived insights primarily from ICU patients who reportedly struggle to recall and reflect upon their experiences (Ethier et al., 2011). Therefore, in this study, we focus on the perceived experiences of healthcare professionals (HCPs) who have frequent and collective interactions with patients and explore how technology could support creating a healing environment that provides holistic and optimal care for ICU patients.

## Phase I: Understanding ICU Patient Experience From the Perspective of HCPs

## Method

### Study design, Participants, and Settings

To explore the role of the ICU environment on patient well-being, we designed a mixed-method study consisting of an online survey and an online semi-structured follow-up interview. The online survey aimed to investigate how current ICU environments support care activities and patient experience and identified factors that positively and negatively contribute to these aspects. A follow-up interview was carried out to gain a more in-depth understanding of patient needs and challenges experienced by HCPs in relation to different care activities. All methods described in this section were approved by the Ethics Committee of the University of Twente, the Netherlands (reference number: 2021.110).

HCPs from four Dutch hospitals (two academic and two nonacademic) participated in the study. A total of 27 HCPs completed the online survey: 13 from academic hospitals and 14 from nonacademic hospitals. All HCPs work in adult ICUs including medical and surgical, thorax, and cardiothoracic ICUs. The majority of HCP participants were ICU nurses (*n* = 22) followed by intensivists (*n* = 4), and anesthesiologists (*n* = 1). Most participants had more than 10 years of experience (*n* = 19, 70%). A total of six of 27 survey participants took part in the follow-up interview. These were five ICU nurses (two from academic and three from nonacademic hospitals) and one intensivist (from a nonacademic hospital), all with at least 5 years of experience.

### Procedure

**Figure 1.**
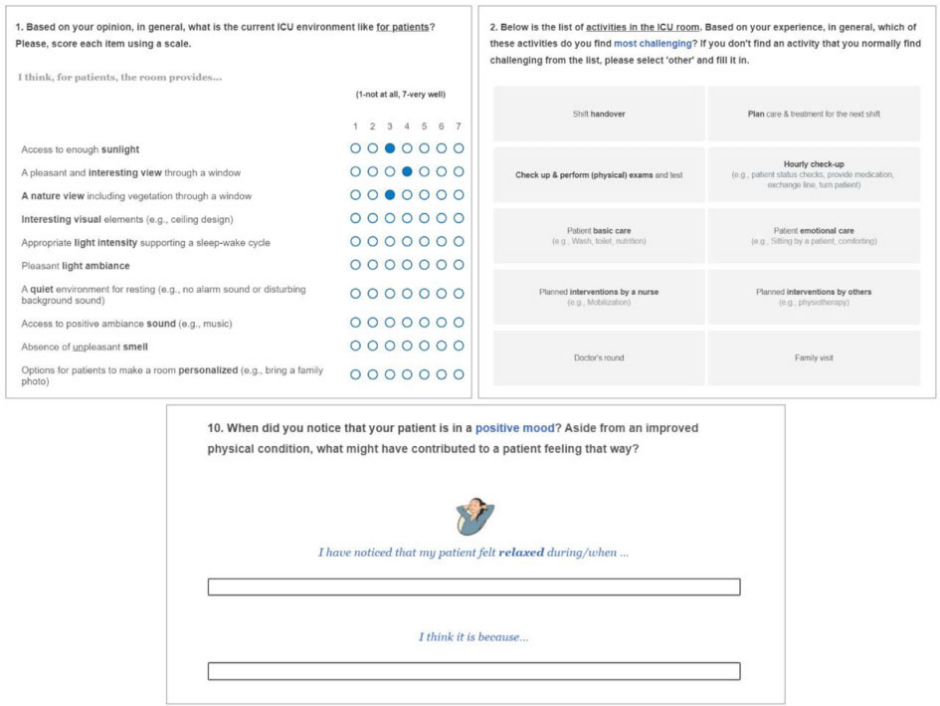
Screenshots of our online survey. *Source*: Illustration used in the survey from the Pick-A-Mood pictorial tool. Used with permission Desmet et al. (2016). Pick-A-Mood Manual. Delft University of Technology, Delft.

The link to the online survey was shared on the internal newsletter of each hospital ICU department. To lower language barriers, participants could choose either an English or Dutch version. Each participant had to provide their consent on the opening page of the online survey before proceeding with the questionnaire. The online survey included both closed- and open-ended questions covering the following topics: the perceived level of environmental support of the ICU on (1) the patient (affective) experience, (2) different care activities, and (3) factors that either positively or negatively contribute to the patient experience. To assess the affective qualities of the ICU, environmental factors contributing to patient experience were derived from healing environment literature (DuBose et al., 2018; Huisman et al., 2012; Simonsen et al., 2022; Verderber et al., 2021). A total of 13 items were selected and used in the questionnaire list. Using this list, HCP participants assessed the perceived level of environmental support in their ICUs on patient experience on a 7-point Likert-type scale and describe the rationales. To understand the influence of the current ICU environment on care activities, we created an overview of ICU activities using the data from our previous observation study with two hospitals participating in the present study. The list of these activities was used to evaluate environmental support in the ICU for patients and HCPs. To explore patient experience, we used a mood measurement tool as mood state can be an indicator of a person’s overall subjective experience (Desmet, 2015). ICU-relevant positive and negative mood states were selected based on patient experience literature (Halvorsen et al., 2022; Krampe et al., 2021) and an adapted version of the mood measurement tool, Pick-A-Mood (PAM; Desmet et al., 2012), was used. HCPs were asked to describe which mood they most frequently see in their patients and what factors contribute to such moods (see Figure 1 for an example question from the online survey). To expand the knowledge of both positive and negative factors contributing to patient experience, HCPs were also asked to describe when patients are in positive and negative moods and what contributed to these moods to their knowledge. The average time spent on the online survey was 15 min.

Participants could indicate whether they were interested in taking the online follow-up interview. Interested participants provided a convenient time to schedule the interview. A 30-min follow-up interview consisted of detailed questions about factors that contribute to the patient experience and related needs.

### Analysis

Online survey data were collected via Qualtrics (www.qualtricsc.com) and the follow-up interview data were recorded and transcribed using Microsoft Teams (www.Microsoft.com). Responses to open-ended questions of the online survey and interview transcripts were systematically analyzed following the principles of reflective thematic analysis (Braun & Clarke, 2006). The relevant data were organized and analyzed using open, axial, and selective coding techniques. First, codes were made by the first author and reviewed and discussed with the second author. These codes captured information relevant to the factors contributing to the entire positive or negative experiences of patients. An agreement on the code was reached by an iterative analysis process. Finally, codes related to the same concept were grouped into categories and themes were identified.

## Results

### Environmental Support for Care Activities in the ICU

The majority of the HCPs perceives emotional care (e.g., sitting with patients, comfort talk) as the most challenging care activity (*n* = 9) followed by dealing with emergency situations (*n* = 6) and performing planned intervention by a nurse (e.g., mobilization; *n* = 4; see Table 1).

**Table 1.** Most Challenging Intensive Care Unit (ICU) Activities According to Healthcare Professionals (HCPs).

Activities of HCPs Take Place in the ICU Room	# HCPs (*n* = 27)
Shift handover	2
Plan care and treatment for the next shift	1
Checkup and perform (physical) exams and test	0
Hourly checkup (*patient status checks, providing meds, exchanging line, taking samples, turning patients, etc.*)	0
Patient basic care (*wash, toilet, nutrition, etc.*)	0
Patient emotional care (*sit by a patient, comfort talk, etc.*)	9
Planned interventions by a nurse (*mobilization, etc.*)	4
Planned interventions by others (*physiotherapy session, etc.*)	3
Doctor’s round	0
Family visit	2
Dealing with emergency situations	6

Both ICU nurses and intensivists most frequently mentioned lack of time as a reason for emotional care being challenging: “Because of shortage of staff and time pressure, there is not always time to provide emotional support even though I think it is really important for my patients” (P1, Nurse). “All other activities occur at a specific moment during the shift. For doctors, there is no indicated time for emotional care. It is a challenge to make room for that” (P3, Intensivist). Also, providing patient emotional care was challenging because of the lack of a proper environment such as “Many interruptions and hostile environment” (P6, Nurse). Lack of privacy was another factor: “There is little personal space for the patient and his or her emotions (in a multipatient room). If you want to separate the bed in the room, your only option is to close the curtains” (P5, Nurse). Lastly, difficulties in communication were mentioned: “Because of their inability to speak, it’s not clear whether the patient understands what I am saying” (P1, Nurse). Importantly, although the environment could play an important role in fulfilling the emotional needs of patients, the results showed that the current ICU fails to convey affective qualities as all scores remain below 3 of 7 (e.g., home-like and inviting; see Table 2).

**Table 2.** Current Impression of the Intensive Care Unit in the Perspective of Healthcare Professionals (1 = *Not at All*, 7 = *Very Well*).

Affective Qualities	Mean	Standard Deviation
Relaxing	2.44	1.26
Interesting	2.44	1.20
Home-like	1.96	1.20
Warm atmosphere	2.56	1.57
Inviting	2.30	1.36

***“Because of shortage of staff and time pressure, there is not always time to provide emotional support even though I think it is really important for my patients” (P1, Nurse).***

***“All other activities occur at a specific moment during the shift. For doctors, there is no indicated time for emotional care. It is a challenge to make room for that” (P3, Intensivist).***

The scores of positive environmental stimuli (Table 3) show how the current ICUs in general lack positive stimuli such as an interesting view, nature view, positive ambiance sound, and interesting (interior) design elements while abounding with elements that can interfere with emotional care for patients: “There is a window in every patient room, but you can only see the wall of the hospital building” (P6, Nurse), “Our ceiling is just white” (P5, Nurse), and “The room is filled with different kinds of clinical equipment. They are so visible and make a lot of noise. It’s far from a homely feeling” (P2, Nurse).

**Table 3.** Positive Environmental Factors in the Intensive Care Unit Grouped into Five Themes (1 = *Not at All*, 7 = *Very Well*).

Positive Environmental Factors	Mean	Standard Deviation
*Light*		
Access to enough sunlight	4.70	1.72
Appropriate light intensity	3.89	1.47
Pleasant light ambiance	3.19	1.33
*Look (view)*		
Interesting (window) view	3.37	1.89
Nature (window) view	2.81	1.61
Interesting visual elements/design	2.48	1.45
Elements of nature	2.37	1.42
*Sound*		
Quiet environment	2.19	1.54
Positive ambiance sound	2.89	1.75
*Smell*		
Absence of unpleasant smell	3.59	1.28
*Other*		
Elements for reorientation	4.78	1.13
Elements for companionship	3.93	1.36
Personalized options	5.04	1.07

***“There is a window in every patient room, but you can only see the wall of the hospital building” (P6, Nurse).***

Table 4 describes how HCPs perceived environmental support for diverse patient activities in the ICUs, which we divided into “cure activities” and “care activities.” Cure activities are directed toward treating the patient’s illness or condition, such as planned interventions. The results showed that the current ICU environment effectively supports cure activities, scoring high in both medical checking (mean = 5.41, *SD* = 1.28) and planned intervention (mean = 4.22, *SD* = 1.45). On the other hand, the current ICU environment shows inadequate support for care activities, which are aimed at enhancing patients’ overall well-being and comfort, especially activities aimed at restoration including resting (mean = 2.70, *SD* = 1.18) and night sleep (mean = 3.04, *SD* = 1.26). “There is always the sound of monitors, ventilators, and talking that are keeping patients awake” (P2, Nurse). “There is too much light (for patients to sleep). Lights are switched on when we enter the room” (P1, Nurse).

**Table 4.** Environmental Support of the Intensive Care Unit (ICU) for Patient Activities (1 = *Not at All*, 7 = *Very Well*).

Patient Activities in the ICU Patient Room	Mean	Standard Deviation
*Care activities*		
Night sleep	3.04	1.26
Resting	2.70	1.18
Entertainment activity	3.30	1.24
Social activity	4.15	1.33
Toileting	3.26	1.76
Washing	4.11	1.75
*Cure activities*		
Planned intervention	4.22	1.45
Medical checking	5.41	1.28

### ICU Patient Experience and Contributing Factors

According to HCP’s, ICU patients predominantly experience negative mood states: tense (*n* = 11, 42%), sad (*n* = 7, 27%), and bored (*n* = 3, 12%). Nine factors contributing to the patients’ negative mood were identified from the analysis of the open-ended questions in the online survey and interview. These factors were categorized into two groups: psychological and physical factors. Table 5 presents an overview of these factors with the quotes from HCPs and the number of mentions.

**Table 5.** Nine Factors Contributing to Negative Intensive Care Unit Patient Experiences and Their Subcategories With Example Quotes From Healthcare Professionals and the Number of Mentions.

Factors	Quotes	Count
Psychological factors		
1. Negative prospect (hopelessness)		22
Concerns about the severity of the current medical condition (17)	“My patient had acute leukemia, There is no nice way to deal with it.”; “When they have to say goodbye to loved ones.”	
Being pessimistic about the future (5)	“They are laying there for a long time without prospect.”	
2. Loss of control (being dependent)		17
Reduced cognitive ability (being confused/not knowing time and place) (6)	“Patients often feel confused when they wake up after sedation.”; “He didn’t know where he was because of reduced brain activity.”	
Lack of information on progress and outcome (4)	“There is no clear information for patients.”; “Patients experience uncertainty about their own body such as what kind of prognosis in the short and long term.”	
Reduced physical ability (4)	“Because of their medical situation, mostly they are not able to do what they want.”	
Communication barrier (3)	“Especially when they are on a ventilator, they are frustrated because it’s uncomfortable and hard to express what they are feeling.”	
3. Traumatic experiences (fear of pain, trauma, hallucination, and delirium)	“I think the fear is the most difficult for ICU patients. When you hear their stories about nightmares and hallucinations, what they see is very frightful.”; “They feel anxious and tense because they are afraid of pain. Not only when they are in pain but also worrying about experiencing it again.”	12
4. Lack of distraction (having nothing to do, depressing environment)	“It’s all white and no warm colors or anything.”; “If they want to look outside, all they see is the building next to the hospital, so it’s not really interesting.”	7
5. Loneliness and disconnectedness	“When patients are getting better, the only thing in their head is how is it at home. (…) they cannot ask their child how things are whenever they want to. It is not easy for patients to pick up the phone and ask their loved ones, and they could be tense because of that.”	5
6. Loss of dignity	“Patients would feel embarrassed with nurses. Nurses don’t mind because it is their daily job. But for patients, it is not their daily job to release their stool while they are lying in bed.”	1
Physical factors		
7. Pain and discomfort		26
(General) pain and discomfort (20)	“Just lying in one position for a long time, it can be very uncomfortable and painful. And every tube and catheter they have in the urinary bladder, in mouth, nasogastric tubes through the nose, the drips in their arms, and bandages around their drips are all sources of discomfort.”	
Painful intervention/care (3)	“If I get it (the way patients are mobilized), I would scream. It’s painful for patients.”	
Discomfort from reduced sedation (2)	“Patients have their most pain in the last days of their ICU admission. Because they are often sedated before, and in their last days of ICU, they are awake and aware of everything.”	
Discomfort from being awake while on a ventilator (1)	“Being on a ventilator is very uncomfortable.”	
8. Illness and exhaustion		8
Illness (6)	“Because they are very sick.”	
Physically challenging interventions (2)	“For patients, it feels like they are running a marathon every day. They have to work, work, work, then finally they can rest. Sometimes they are sad and unhappy that they have to get out of bed and have to do things.”	
9. Lack of sleep and disturbing environment	“The ventilator always makes a sound. You have the noise with every breath you take, it’s quite a lot of noise for 24 hr.”; “The strong beam right above the bed is for us to see what we are doing but probably not nice for patients to sleep.”	5

Convivial toolbox: Generative research for the front end of design.

For the positive patient experience, eight themes were identified and divided into psychological and physical factor groups. Table 6 shows the overview of these themes, quotes from HCPs, and the number of times they are mentioned.

**Table 6.** Eight Factors Contributing to Positive Intensive Care Unit (ICU) Patient Experiences and Their Subcategories With Example Quotes From Healthcare Professionals (HCPs) and the Number of Mentions.

Factors	Quotes	Count
Psychological factors		
1. Human interaction (social support)		17
Interaction with loved ones (11)	*“A visit from grandchildren or their dog brings the most joy in the regular (ICU) life of patients.”*	
Feeling of being cared for (interaction with HCPs) (6)	*“Real contact with either staff or family in peace can have a reassuring effect.”*	
2. Hopeful perspective (anticipation)		8
Sense of getting better (5)	“Patients who cannot move their finger one day lift their hands the next day. It is very rewarding for patients. You can see that patients are very happy with the progress they make.”	
End of ICU stay (3)	*“When they know they can leave the ICU.”*	
3. Distraction from illness		8
Conversation with others (4)	*“When I am in the room and talking to patients, they feel comfortable as there is someone they can talk to and some distraction.”*	
Positive sensory stimulation (3)	*“TV and music help patients to feel less pain.”*	
Change of scenery (being away from ICU) (1)	“When we take patients outside, they get a sense that they are out of bed and out of the ICU. Even if it’s for half an hour, that influences the mindset of patients.”	
4. Sense of safety		7
Being watched by professionals (4)	*“They feel safe when they are getting attention from professionals.”*	
Familiar environment (3)	“It is important for patients to make it more like home. Bringing some nature inside is one way to make it more feel like home.”; “Watching TV helps patients to realize that they are back in the world again. They can see some familiar things from TV.”	
5. Sense of control		6
Being informed about the situation (4)	*“My patients are content when they understand where they are and what the situation they are going through after I explained.”*	
Being able to do what they normally do (2)	*“They are happy when they say what they want because it is closest to what they normally do and provides them a sense of control.”*	
6. Feeling like oneself (dignity)	“It is very important that they can feel protected from people walking in when they are naked and have somebody taking care of their appearance (…). There is a moment when the nurses pay attention to them. We put a patient in a chair with makeup on and that is a very cheerful moment for her.”	3
Physical factors		
7. Feeling restored (good night’s sleep and rest)	*“Everyone who can sleep at night feels better during the day.”*	6
8. Relief from pain and discomfort	*“Feeling less pain.”*	5

## Phase II: Creating a Healing ICU Environment Supporting Patient Well-Being

## Method

### Study Design, Participants, and Setting

A multistakeholder workshop was conducted to explore the potential of ICU environments in optimizing support for patient well-being and possible technology-based solutions. The workshop was built upon the results of the mixed-method study and was designed to include multistakeholders, which resulted in a collaborative workshop where different stakeholders came together to share their knowledge, expertise, and perspectives on a particular topic (Sanders & Stappers, 2012). This approach was adopted as the goal of this workshop is to generate innovative and feasible solutions through a structured process that leverages the diverse perspective and knowledge domains of the stakeholders.

A total of 11 participants were recruited to have a diverse group of participants. The group consisted of four ICU nurses from two Dutch hospitals (participating also in *the study Phase 1*), three academic researchers in healthcare design and psychology, and four industry experts in health technology specialized in the critical care domain.

### Procedure

**Figure 2.**
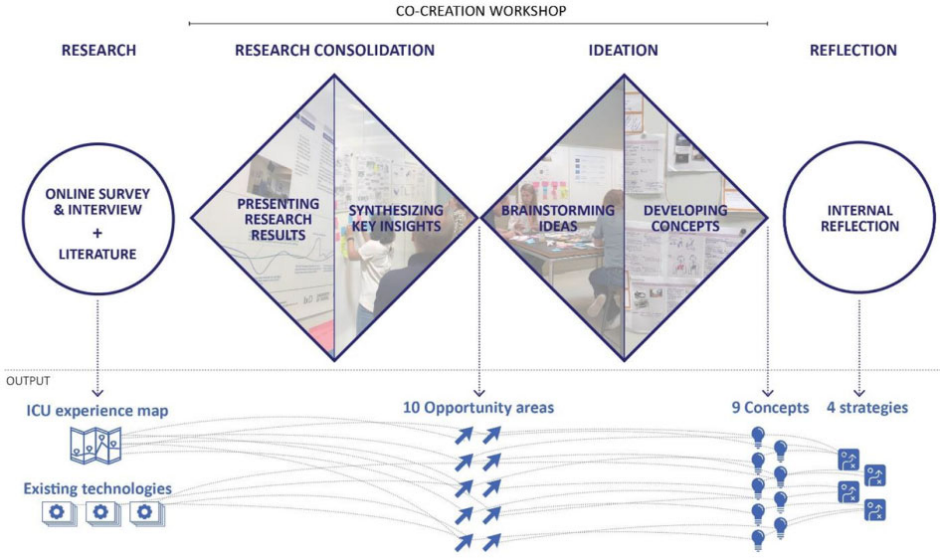
Overview of the workshop.

The co-creation workshop was designed following the double diamond model (Design Council, 2015). The workshop consisted of two sessions: consolidation and ideation. The entire workshop lasted 4.5 hr taking 2 hr for the first session and 2.5 hr for the second. Figure 2 illustrates the overall structure of the workshop next to its input and output.

In the first phase of the workshop, the research consolidation session, a journey map and a visual summary of the online survey results (the content of Tables 5 and 6), and an overview of a literature review on technology-based interventions used in the ICU (Kim et al., 2021) were presented. During the presentation of the data, participants were encouraged to make notes on what they considered as key insights on post-its. Next, these collected key insights were synthesized and summarized as opportunity areas through group discussion. For the second phase of the workshop, the ideation session, participants worked in pairs and developed a vision statement (e.g., “I want the ICU to be….”) based on the opportunity areas and brainstormed about technological solutions. A set of inspirational images and drawing materials were provided with a template containing questions that could support detailing the ideas such as “What problem does it solve?” and “How does it work?” During the last 30 min, each duo presented the vision and ideas they created to the group for feedback.

### Analysis

The workshop data, including opportunity areas that were identified and vision statements and initial concepts generated during brainstorming, were analyzed to extract actionable design criteria and opportunities. By integrating them, four strategies were developed by the first author and reviewed with the coauthors. We will further elaborate on these strategies in the Results section.

## Results

The consolidation session yielded 10 opportunity areas including *address the emotional needs of patients*, *support patient control*, and *provide a home-like environment* (see Table A1 in the appendix for all identified opportunity areas). The ideation session used these opportunity areas and participants generated a total of nine initial concepts which include a *multimodal calm wake-up experience*, a *digital bridge to home*, and a *digital healing journey map* (see Table 7 for a detailed description of these three examples, also see Table A2 in the appendix for all nine concepts). All concepts were presented in the group and their benefits, challenges, and execution plan were discussed. These concepts were presented on a large board which allows an overview and easy comparison between concepts for participants.

**Table 7.** Examples of Concepts Generated During the Ideation Session.

Concept	Multimodal Calm Wake-Up Experience	Digital Bridge to Home	Digital Healing Journey Map
Opportunity area	Address emotional needs Support patient control	Address emotional needs Provide a home-like environment	Address emotional needs Support patient control Provide information
Concept description	The concept creates a multimodal experience for patients to wake up after sedation in a calm and pleasant manner, rather than feeling disoriented and fearful. This experience incorporates prerecorded voice messages and photos from family members. It features soft, gradually dimming lighting reminiscent of sunrise through immersive projection mapping, accompanied by ambient audio devices.	The concept supports patients in having their everyday experiences in a familiar environment, such as eating, watching TV, playing games, and listening to music, by connecting the ICU to their home using virtual reality (VR), augmented reality (AR) and screen devices.	The concept provides a real-time overview of a long-term treatment plan and updates. The visual of a boat sailing along the river represents the healing journey of patients. The distance traveled by boat and the obstacles passed represent the progress made by patients. The visual can be provided using ambient displays or handheld devices.
Visuals supporting concepts created by participants	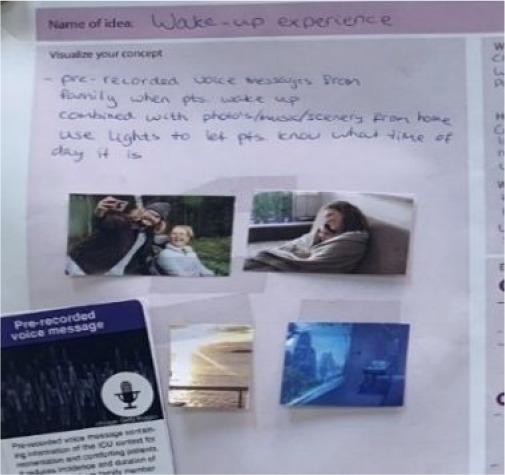	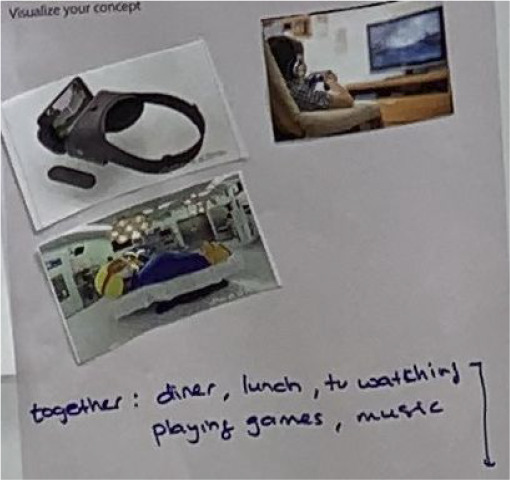	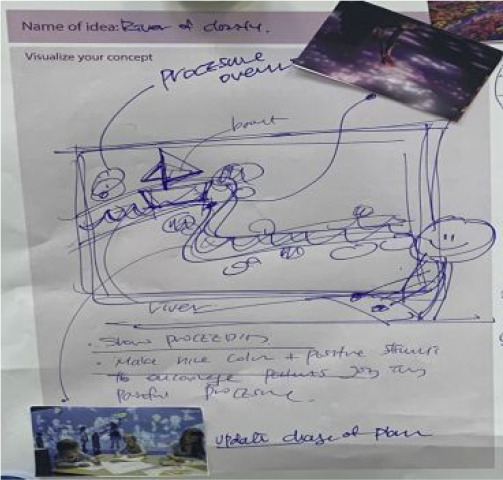

### Four Strategies to Create Technology-Enabled Healing ICU Environment

The concepts developed by participants provide concrete examples of how technologies could support creating healing ICU environments. Our aim is, for these findings, to enable other designers and HCPs to create solutions that are customized to their unique ICU contexts. To achieve this, we looked into the values underlying each concept and synthesized them into strategies that comprehensively address patient experience factors (see Table 8).

**Table 8.** Four Strategies to Create Technology-Enabled Healing Environments With Their Associated Factors and Design Concepts.

Strategy	Associated Factor	Related Concept
1. Make the clinical journey and progress visible to patients	Hopeful perspective Sense of control	Digital healing journey My progress diary
2. Connect to home and loved ones	Human interaction Sense of safety Distraction from illness	Digital family platform Digital bridge to home Virtual home
3. Create calm wake-up and ready-for-sleep experiences	Feeling restored (good night’s sleep and rest) Sense of safety Sense of control	Multimodal calm wake-up experience Good night ICU protocol and ambiance experience
4. Personalize positive sensory distractions	Distraction from illness Sense of control	Personalized digital window Adaptive ICU

*Strategy 1. Make the clinical journey and progress visible to patients*: The ICU environment can promote a sense of control and a hopeful perspective among ICU patients by providing an overview of the medical journey which gives patients an idea of what they went through and what to expect for the coming period. To encourage patients, the small progress that patients make should be emphasized in an overview. It could positively influence the mindset of patients and support them in building anticipation toward recovery in the ICU context, where the path to discharge often appears distant and pessimism can easily set in.
*Strategy 2. Connect to home and loved ones*: Interactions with loved ones promote a sense of safety, human interaction, and distraction from illness for patients. Family visits, for instance, are among the most positive activities in the ICUs that alleviate the loneliness and boredom of patients while providing reassurance. To optimize the benefits of interactions with loved ones, an ICU environment should support pleasant family visits by providing relaxing surroundings and also by supporting pleasant and continuous interactions between patients and loved ones outside of family visit time.
*Strategy 3. Create calm wake-up and ready-for-sleep experiences*: When waking up from sedation, ICU patients often suffer from feelings of being lost, fear, and anxiety due to an unfamiliar environment. The hostile ICU environment also disturbs patient sleep. The ICU environment can promote the restoration of patients (good night’s sleep and rest) by creating an ambiance that accommodates timely needs and gives patients a sense of familiarity and reassurance.
*Strategy 4. Personalize positive sensory distractions:* ICU patients need positive sensory stimulations that can distract them from pain and negative thoughts. Using ambient technology including ambient light, display, and sound, the ICU environment can provide a wide range of positive stimulations that cater to the different emotional needs of patients. For instance, to create distractions by positive stimuli, the level of stimuli can be adjusted depending on the perceived pain and discomfort level of patients. To address dynamic negative emotional experiences that ICU patients go through, such as loneliness, sadness, fear, and anxiety, different types of positive stimulation could be provided. For instance, to alleviate fear and anxiety, calming and reassuring stimuli can be utilized, while uplifting stimuli can help alleviate sadness.

## Discussion and Conclusion

In this study, we explored the role of the ICU environment in promoting patient well-being from the perspective of HCPs and envisioned how technology could support creating healing ICU environments. The mixed-method study (Phase I) yielded insights into the ICU patient experience including positive and negative contributing factors as well as current limitations and challenges. The co-creation workshop (Phase II) resulted in identifying key areas of improvement and developing concepts that support creating an ideal environment for patients. Drawing from these insights, we formulated four strategies that harness the potential of technology in enhancing ICU patients’ well-being.

An interesting finding from the first phase of the study was that most negative factors stem from *being in the ICU environment* (e.g., loss of control, lack of distraction, loneliness, and disconnection) than patients’ illness itself (e.g., pain, discomfort, and negative perspective). These negative factors mentioned by HCPs are aligned with the findings from studies conducted with ICU patients (Abuatiq, 2015; Halvorsen et al., 2022; Krampe et al., 2021; Rose et al., 2014) which include loss of control, loneliness, (dull) design of the room, as well as pain and functional distress. We also found that most of these factors are intertwined and can create chain effects; not addressing one negative factor can activate another negative factor. Taking an example, the lack of distraction can increase subjective pain which disturbs sleep and lead to worsening health outcomes and recovery. Therefore, instead of addressing individual factors in isolation, adopting a more holistic approach that encompasses most negative factors would be more effective. Previous studies (Halvorsel et al., 2020; Kim et al., 2021) also support the importance of addressing multidimensional needs in the ICU to promote patient well-being. Our findings on positive factors inform how patient needs are currently met in the ICU environments from the perspective of HCPs. Most of these factors correspond with findings from other studies conducted with ICU patients (Aro et al., 2012; Halvorsen et al., 2022; Van Keer et al., 2017) including social support, sense of safety, sense of control, distraction from illness, as well as relief from pain and discomfort. Importantly, we found that most positive factors are currently reliant on HCPs who cannot act on these needs due to the high workload in the current ICUs. Studies (Carayon & Alvarado, 2007; Hugonnet et al., 2007) pointed out the workload of HCP as a key challenge in the ICU, which leads to “invisible tasks,” such as emotional care, being left unattended (Halvorsen et al., 2022; Kitson, 2018). This problem resonates with our findings: Patient emotional care is currently the most challenging aspect in the ICU for HCPs. We found that despite the effect of positive environmental factors on patient well-being, their implementation is limited or absent. This limitation might be due to physical constraints, such as the location or architectural structure of the hospital building, which do not allow access to nature views or systemic constraints, for instance, not considering positive elements in the hospital interior design process.

***We also found that most of these factors are intertwined and can create chain effects; not addressing one negative factor can activate another negative factor.***

***Importantly, we found that most positive factors are currently reliant on HCPs who cannot act on these needs due to the high workload in the current ICUs.***

The results of the second part of the study provided rich insights into how technology can act upon the current limitations of ICU environments. The four strategies we introduced based on the results from an extensive stakeholder workshop are in line with nonpharmacological interventions that are currently practiced in the ICU. These include, for example, the provision of sufficient information, family involvement, and personalized care from the ICU ABCDEF bundle (Marra et al., 2017) and the hospital elder life program (HELP; Zachary et al., 2020). Along with the strategies, our concepts showed how to extend the implementation of these strategies by adopting technologies. Most technologies are informed by existing technology-based interventions that are also evidence-based. For instance, personalized audio stimuli (Cheong et al., 2016) and the provision of visualized information about medical procedures (Ryu et al., 2019) proved to reduce anxiety among ICU patients.

Our study results contribute to the knowledge of the current positive and negative factors of ICU patient well-being from the perspective of HCPs. We showcased how technology could enable holistic and personalized care in the ICU environment with a variety of concepts and four strategies. We expect our findings to inform designers and developers of healthcare technologies and HCPs in creating customized interventions adapted to their ICU contexts.

***We showcased how technology could enable holistic and personalized care in the ICU environment with a variety of concepts and four strategies.***

## Limitations and Future Directions

Our study provided valuable insights from the perspective of HCPs. This study was conducted with ICUs located in the Netherlands. Considering the differences in ICUs with other countries that might affect the perceived experiences of patients, the findings may not be readily transferrable to ICUs in other countries. While most of our insights are supported by other study findings conducted with patients, the development of concepts and strategies for enhancing patient well-being was derived from the ideas of HCPs. Furthermore, the current sample size (*n* = 27) has offered valuable insights. However, due to our approach (i.e., distributing an online survey using an anonymous link), we cannot rule out a sampling bias in the absence of a precise response rate. That is, participation in our study might reflect interest in the topic of our research. Hence, to validate and extend these findings, follow-up studies with a larger sample size and an appropriate response rate are warranted. To fully explore and validate the identified opportunities for enhancing the ICU environment, further validation with ICU survivors is required. The scope of our study is enhancing ICU patient well-being on an experiential level. Hence, the four strategies we developed do not address problems on an organizational or systemic level. However, considering that the successful implementation of technology-based solutions goes hand in hand with systemic changes, such as changes in procedures and physical aspects of interior design, future studies will need to include multiple perspectives from diverse stakeholders, including ICU patients. We encourage future studies to extend and detail our strategies by involving a wide range of stakeholders.

## Implications for Practice

- Overall, this study presents how digital technology can extend the potential of the ICU environment in supporting patient well-being from the perspective of HCPs.
- The first part of the study outlines both positive and negative factors influencing patient experiences in the ICUs and the challenges experienced by HCPs during care activities, which highlights key areas for digital technology to address in order to enhance ICU patient well-being.
- The second part of the study proposes four strategies that provide guidance on how to adopt technology in the ICU environment to offer holistic and optional care: providing clear information for patients and family, establishing connections to home and loved ones, creating an ambiance conducive to effective rest, and offering personalized distractions.

## Appendix

**Table A1.** Identified Opportunity Areas and Examples Opportunity Areas Examples.

1. Address the emotional needs of patients	*Alleviate fear and anxiety*
2. Promote a good night’s sleep	*Promote relaxation and provide a quiet environment*
3. Provide a home-like environment	*Include more personalized factors and make sure positive elements such as family photos and cards are reachable for patients*
4. Provide continuous care and monitoring	*Patients need more care during the night when logistically it is not possible*
5. Support patients control	*Support patient communication and reduce dependency*
6. Provide information	*Make progress visible and support patients celebrate it*
7. Provide a quiet moment	*Control crowdedness and provide personal space*
8. Promote enjoyable personal interactions	
9. Promote pleasurable family visits	*Instruction for how to act and communicate with patients*
10. Differentiate treatment depending on a patient’s profile	*Personalized pain management*

**Table A2.** A Summary of the Concepts Generated During the Multistakeholder Co-Creation Session.

Concept	Description
My progress diary	The concept offers patients an overview of the progress they have made and celebrates each step toward discharge through straightforward and easy-to-understand visual representations. This progress diary can also be utilized by patients’ family members to stay informed about the patient’s status and to provide encouragement by celebrating their small progress together.
Digital family platform	The concept offers a digital platform where the patients’ status is regularly updated for family members. Additionally, family members can upload photos and voice messages for the patients. By offering current information and familiar elements, this concept promotes a sense of being connected, alleviating tension and anxiety for both patients and their family members.
Personalized digital window	The digital window concept offers landscapes, dynamic lighting, and soundscapes that are tailored to the specific stages of patients’ recovery processes.
Virtual home	The artificial window concept delivers multisensory stimuli, including visuals and sounds reminiscent of patients’ own home environment or other places where they find comfort and familiarity.
Adaptive ICU	The concept creates an ambiance that corresponds to the physical and psychological needs of patients throughout the day in the ICU, such as energizing during physiotherapy sessions and calming down for effective rest.
Good night ICU protocol and ambiance experience	The concept supports patients to physically and psychologically be prepared for better sleep through a protocol combined with activities (activities during daytime and mind-clearing before sleep) and ambient experience supporting these activities.
